# Euthanasia of laboratory mice: Are isoflurane and sevoflurane real alternatives to carbon dioxide?

**DOI:** 10.1371/journal.pone.0203793

**Published:** 2018-09-10

**Authors:** Nicole Marquardt, Malte Feja, Hana Hünigen, Johanna Plendl, Lena Menken, Heidrun Fink, Bettina Bert

**Affiliations:** 1 Institute of Pharmacology and Toxicology, Department of Veterinary Medicine, Freie Universität Berlin, Berlin, Germany; 2 Institute of Veterinary Anatomy, Department of Veterinary Medicine, Freie Universität Berlin, Berlin, Germany; Indiana University Purdue University at Indianapolis, UNITED STATES

## Abstract

In the European Union (EU) millions of laboratory mice are used and killed for experimental and other scientific purposes each year. Although controversially discussed, the use of carbon dioxide (CO_2_) is still permitted for killing rodents according to the Directive 2010/63/EU. Within the scope of refinement, our aim was to investigate if isoflurane and sevoflurane are an appropriate alternative killing method to CO_2_ in mice. Different concentrations of CO_2_ (filling rates of 20%, 60%, 100%; CO_2_ 20, 60, 100), isoflurane (Iso 2%, 5%) and sevoflurane (Sevo 4.8%, 8%) were compared in two mouse strains (NMRI, C57Bl/6J) using a broad spectrum of behavioral parameters, including the approach-avoidance test, and analyzing blood for stress parameters (glucose, adrenaline, noradrenaline). We focused in our study on the period from the beginning of the gas inlet to loss of consciousness, as during this period animals are able to perceive pain and distress. Our results show that only higher concentrations of CO_2_ (CO_2_ 60, 100) and isoflurane (5%) induced surgical tolerance within 300 s in both strains, with CO_2_ 100 being the fastest acting inhalant anesthetic. The potency of halogenated ethers depended on the mouse strain, with C57Bl/6J being more susceptible than NMRI mice. Behavioral analysis revealed no specific signs of distress, e. g. stress-induced grooming, and pain, i. e. audible vocalizations, for all inhalant gases. However, adrenaline and noradrenaline plasma concentrations were increased, especially in NMRI mice exposed to CO_2_ in high concentrations, whereas we did not observe such increase in animals exposed to isoflurane or sevoflurane. Escape latencies in the approach-avoidance test using C57Bl/6J mice did not differ between the three inhalant gases, however, some animals became recumbent during isoflurane and sevoflurane but not during CO_2_ exposure. The rise in catecholamine concentrations suggests that CO_2_ exposure might be linked to a higher stress response compared to isoflurane and sevoflurane exposure, although we did not observe a behavioral correlate for that. Follow-up studies investigating other fast-acting stress hormones and central anxiety circuits are needed to confirm our findings.

## Introduction

The fate of almost all experimental animals is being killed at certain stages of each study, either to gain blood, tissue and other specimens or at humane endpoints to prevent any extension of stress or pain. Furthermore, during the process of generating transgenic strains, redundant animals lacking the required genetic background are killed.

More than 50 years ago, the concept of the 3Rs (replace, reduce, refine) in animal experimentation has been promoted [1]. Refinement within the process of killing laboratory animals means to use the method that causes the least minimum of pain, suffering, and distress [2]. The Directive 2010/63/EU on the protection of animals used for scientific purposes stipulates that a competent person shall carry out killing using a method that is appropriate for respective species [3]. In Annex IV of the Directive, the exposure to carbon dioxide (CO_2_) by gradually filling is suggested as an adequate method for killing laboratory rodents. Inhalation of CO_2_ is still the most common euthanasia method because it rapidly and reliably induces loss of consciousness with minimal safety concerns for the user [4]. Several authors regard CO_2_ as an appropriate killing method for rats [5, 6] and mice [7–10] under certain circumstances (e. g. appropriate CO_2_ concentration, specific surroundings like habituation to the chamber; for review see also [4, 11]).

However, the use of CO_2_ for euthanizing animals has been questioned as studies have shown that the exposure to CO_2_ causes aversion in rats and mice [12, 13], demonstrated by several behavioral tests, such as the preference test [14–17], the approach-avoidance test [18–21], and the aversion-avoidance test [22]. Moreover, inhalation of CO_2_ concentrations of approximately 50% are perceived as unpleasant and painful in most humans [23, 24], provoking a feeling of breathlessness, whose quality and quantity correlates positively to the partial pressure of CO_2_ in the blood [25, 26]. It is thought that CO_2_ diffuses into the mucosal cells of the respiratory tract and decreases the intracellular pH by reacting with water, which may selectively excite primary afferent nociceptors [27, 28]. It has been shown that intranasal application of CO_2_ in rats dose- and time-dependently activates pain-related neurons in the medullary dorsal horn [29]. In addition to the behavioral findings, mice and rats killed with CO_2_ (prefilled or gradually filled) show several histopathologic alterations of the lung, such as hemorrhage and perivascular edema [7, 24, 30, 31].

Hence, assessment and development of alternatives to CO_2_ killing has become a focal point in laboratory animal science. Recent studies recommend the use of volatile anesthetics for euthanasia [12, 13, 22], but it still needs to be clarified whether they cause less pain, distress, and suffering compared to CO_2_. In several behavioral tests, halogenated ethers such as isoflurane, sevoflurane, enflurane, and desflurane as well as halothane were found to be less aversive than CO_2_ in mice and rats [12, 15, 17, 22]. However, in case of isoflurane there is also evidence that multiple exposures lead to increased aversiveness in naïve rats compared to the first exposure [22]. Sevoflurane is reported to be less irritant in humans than isoflurane [32, 33], and is therefore used in pediatric patients with maximum vapor concentration for mask induction of narcosis [34]. A recent study also described sevoflurane to be the least aversive gas in mice in comparison to isoflurane and CO_2_ [35]. However, there is also evidence that subanesthetic concentrations of isoflurane and sevoflurane decrease the respiratory response provoked by hypercapnia in humans [36] and mice [37].

Only few studies specifically compare the exposure to CO_2_ with the exposure to other inhalant anesthetics in rodents, and most of them do not focus on the humaneness of the method. Isoflurane rather than CO_2_ is recommended as an anesthesia method for blood sampling in rats, since the heart rate and blood pressure of the animals are affected by CO_2_ for a longer time post-anesthesia than by isoflurane, although the authors did not further substantiate this observation [38]. Increased blood corticosterone concentrations have been observed after isoflurane and CO_2_ exposure, which were significantly higher after repeated administration of CO_2_ [39]. Another study comparing the effects of CO_2_, methoxyflurane, ether, and isoflurane on different blood parameters, recommended CO_2_ as a suitable method for retro-orbital blood sampling because of its smooth recovery period [40]. Valentine et al. suggested that anesthetizing mice with isoflurane before CO_2_ euthanasia actually increases behavioral and neuromolecular signs of stress in comparison to killing with CO_2_ alone [41].

As the present data does not conclusively demonstrate the advantages of halogenated ethers over CO_2_, the aim of our study was to evaluate comprehensively the humaneness of isoflurane and sevoflurane as killing methods in mice, and to compare their effects to those of CO_2_. During induction of anesthesia, animals are able to perceive pain, distress, and suffering until they are unconscious, which refers in general to the end of stage 1 of anesthesia. Although the appropriate surrogate measure for unconsciousness in rodents is not finally defined, the loss of righting reflex (LORR) has been described as a first indicator of insensibility and as a good correlate for the loss of consciousness in humans [42]. The subsequent loss of the pedal withdrawal reflex (LOPR) is a more conservative measure for surgical tolerance [13, 43]. Hence, we focused the behavioral analysis part of our study on the period from the beginning of gas exposure to the LORR. In the first part of the study (Experiment 1), the three inhalant gases were applied in different concentrations by gradual filling and the behavior was extensively analyzed on the basis of the assessment procedure for the evaluation of drug effects described by Irwin [44]. The behavioral analysis included the recording of ultrasound and audible vocalization, the latter a specific sign of acute pain [45, 46]. In general, mice produce ultrasonic vocalizations during nonaggressive social interactions (for review [47]), whereas acute pain leads to specific audible vocalizations [48]. Additionally, we registered the latencies to the onsets of LORR and LOPR as time is an important factor for a daily routine of euthanizing animals. For humane reasons, blood samples for catecholamine and glucose concentrations were collected after the LOPR had been achieved. As increases in plasma catecholamine concentrations precede corticosterone responses to stress in rats [49], we decided to measure plasma adrenaline and noradrenaline concentrations as faster responding stress markers. However, it has to be stated that the absolute time points for plasma catecholamine and corticosterone concentrations to peak are difficult to determine. Blood glucose concentrations were also recorded as possible additional stress marker as a rapid and marked increase in plasma glucose levels has been associated with the stress of ether anesthesia in two mouse strains [50]. The respiratory tract was examined for macroscopically visible alterations and was subsequently prepared for further histological analysis. Since strain differences have been already described for the effects of CO_2_ (see [51]), we conducted our experiments in two commonly used mouse strains, i. e. NMRI and C57Bl/6J mice.

In the second part of our study (Experiment 2), we investigated the aversiveness of CO_2_, isoflurane, and sevoflurane in an approach-avoidance test according to the protocol by Makowska et al. [52]. Therefore, C57Bl/6J mice were exposed to isoflurane, sevoflurane, or CO_2_ in three ascending concentrations to determine whether one gas is more avoided than the other.

## Methods

### Experimental animals

The animals used in this study, male and female NMRI (HsdWin:NMRI) as well as male and female C57Bl/6J (C57BL/6JOlaHsd) mice originally obtained by Harlan Laboratories (Netherlands), were control mice of previous behavioral experiments conducted at our institute or surplus mice of our own breeding. These mice had been either untreated or had only received saline injections in previous tests. In order to reflect the conditions in a normal laboratory, the age of the animals covered a broad spectrum as 8–22 weeks for NMRI mice and 10–23 weeks for C57Bl/6J mice. Male mice weighed 37.4 ± 3.8 g (NMRI) and 27.6 ± 2.3 g (C57Bl/6J), female mice 30.8 ± 3.6 g (NMRI) and 20.8 ± 2.0 g (C57Bl/6J).

Before the actual experiments started, mice were group-housed by gender (max. 4 animals per group) in Makrolon type III cages (420 x 265 x 150 mm, floor area 825 cm^2^, Bioscape, Wandlitz, Germany) with dust-free wooden bedding (ssniff^®^ Lignocell 3–4 S, Soest, Germany). Cage enrichment included cellulose and cardboard tubes as nesting material and sunflower seeds in the bedding to encourage seeking behavior. The animals were fed a pelleted mouse diet (ssniff^®^ EF R/M-H 10 mm, ssniff Spezialdiäten GmbH, Soest, Germany) ad libitum and had free access to tap water. The light/dark cycle in the rooms was 12/12 h (lights on at 5:00 a. m.) with artificial light (~100 Lux in the cage). The room temperature was 23 ± 1°C, with a relative humidity of 50 ± 10%. Animals were handled and weighed at least once a week. Once weekly, home cages were cleaned and equipped with new bedding by a professional animal keeper. Animals were free of pathogens according to FELASA recommendations and health status was monitored quarterly by using sentinel animals.

The experiments were performed in accordance with the German Animal Welfare Act and the corresponding regulation. Breeding, husbandry, and the execution of experimental procedures were approved by the competent authority, i. e. Landesamt für Gesundheit und Soziales (registration numbers ZH5, G 0460/09).

### Experiment 1

#### Narcotic chamber

**Fig 1:**
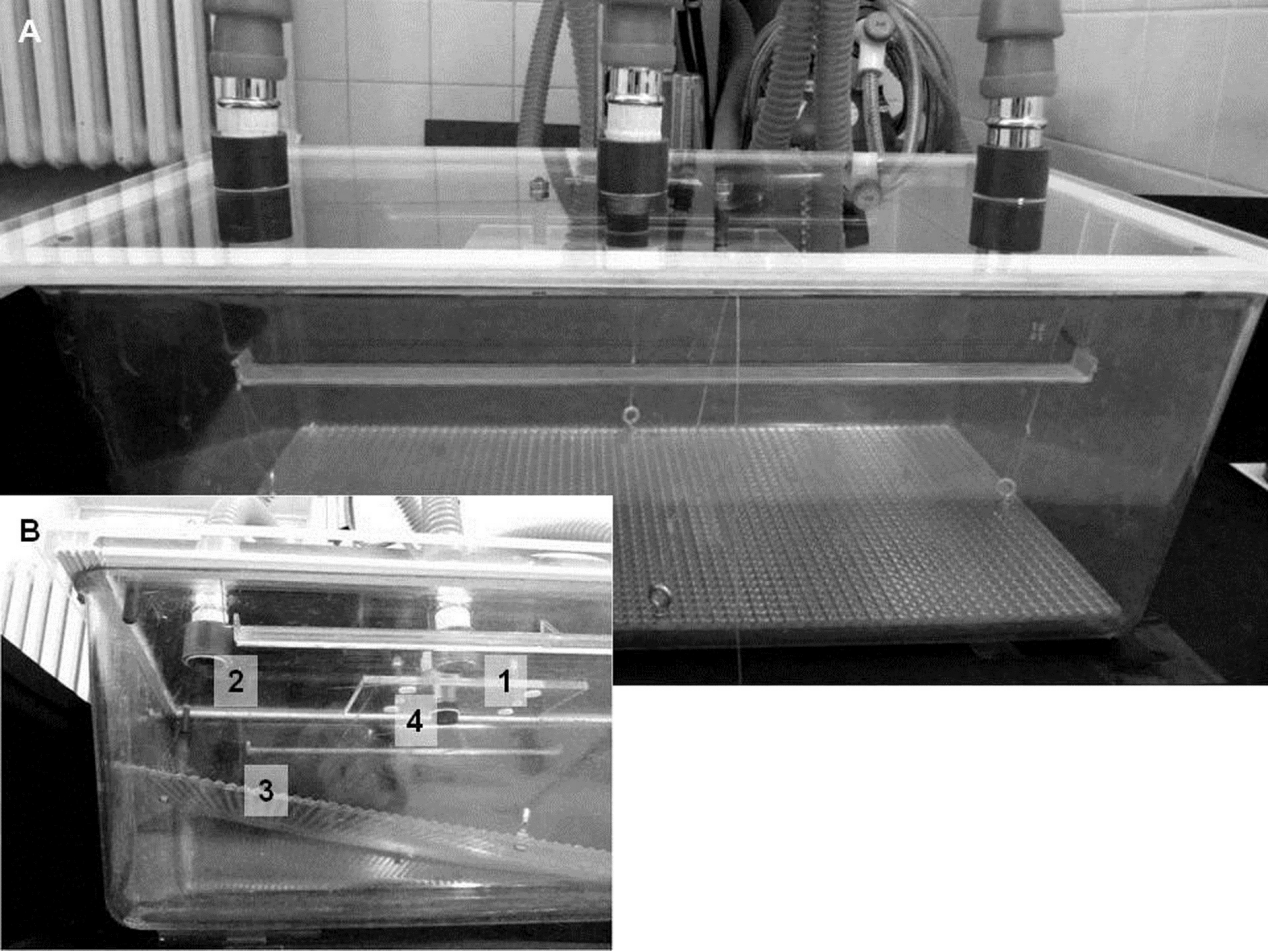
**A.** Narcotic chamber according to Corbach [8] consisting of a Macrolon type III cage and a clear acrylic glass lid. **B.** Detailed view of the narcotic chamber. 1) Connector for gas inlet, with a turbulence device attached below. 2) Connector for gas outlet, with an additional connector on the opposite side. 3) Acrylic glass lid. 4) Microphone.

The narcotic chamber consisted of a Macrolon type III cage (420 x 265 x 150 mm (top), floor area 825 cm^2^, Bioscape, Wandlitz, Germany; volume: 14 l) and a purpose-designed, clear acrylic glass lid according to the thesis by Corbach [8] (Fig 1). Three openings (each 3 cm diameter) were integrated in the lid: two holes at each end for connecting the exhaust tubes and one hole in the center for connecting the anesthetic gas inlet. An acrylic glass square (7 cm x 10 cm x 0.5 cm) was attached 1.8 cm below the center hole as a turbulence device to provide homogenous mixing of the gases [8]. Conventional sealing strip was attached on the bottom of the lid to prevent air exhausting through the edges. In order to avoid the influence of different odors, each animal was assigned to an individual cage. The bottom of the cage contained an acrylic glass board (36.8 cm x 19.8 cm x 0.3 cm) with a rough surface and with 4 thin nylon threads connected to the middle of each side to allow lifting for testing of the righting reflex. An ultrasound sensitive microphone (ultrasound sensor: Knowles, Dover Corporation, Downers Grove, Illinois, USA; plug-in connector: Neutrik^®^ NC MX, Dachau, Germany) was suspended in the center of the test cage via a hole in the lid next to the gas tube hole and connected to the Avisoft Ultrasoundgate 116 (Avisoft Bioacoustics, Glienicke, Germany) and a laptop. Two video cameras (Panasonic^®^ HDC-TM 700, Kadoma Osaka, Japan and Canon PowerShot SX 200 IS, Tokyo, Japan) were positioned close to the test cage to record the behavior from two different angles.

#### Anesthetic apparatus and substances

Eight experimental groups per strain (CO_2_ 20, CO_2_ 60, CO_2_ 100, Iso 2%, Iso 5%, Sevo 4.8%, Sevo 8%, and Air) were tested. A sample size calculation was performed to determine the number of animals to be used: Each group contained 16 animals (8 male and 8 female). All animals were manually assigned to the treatment groups and it was assured that each age group was comparably represented in each treatment group and for each sex. We used CO_2_ from compressed gas cylinders (Air Liquide Deutschland GmbH, Berlin, Germany) fitted with a combined pressure reduction valve and flowmeter. Isoflurane (Forene^®^, Abbott GmbH & Co.KG, Wiesbaden, Germany) and sevoflurane (Sevorane^®^, Abbott GmbH & Co.KG, Wiesbaden, Germany) were delivered via a custom fitted anesthetic machine on the basis of a Dräger Sulla^®^ device (Dräger Medical GmbH, Lübeck, Germany), that allowed the enrichment of compressed air (Air Liquide Deutschland GmbH, Berlin, Germany) with an isoflurane vapor (Dräger Vapor^®^ 19.1 Isoflurane 5%, Lübeck, Germany) or sevoflurane vapor (Dräger Vapor^®^ 19.3 Sevoflurane 8%, Lübeck, Germany) and the direct introduction of the gas into the test cage. One hundred percent CO_2_ was delivered at three different filling rates: 20, 60, and 100% of cage volume per minute (% CV/min, = treatment groups CO_2_ 20, CO_2_ 60, and CO_2_ 100) which corresponds to flow rates of 2.8, 8.4, and 14 l/min, respectively. Isoflurane and sevoflurane were delivered with a constant filling rate of 71% CV/min, which is the maximum rate for both anesthetic agents (10 l/min) to allow for rapid filling of the cage, and with low 1.5 minimum alveolar concentration [53, 54] and maximum setting of the vapors. Two different concentrations of isoflurane and sevoflurane were used: 2% and 5% isoflurane (Iso 2% and Iso 5%) and 4.8% and 8% sevoflurane (Sevo 4.8% and Sevo 8%). Concentrations of the narcotic gases were based on previous studies [53, 54].

An estimation of gas concentration in the test cage was calculated for the time points of the onset of the LORR and the LOPR for each narcotic gas and strain. Assuming that at any given time the narcotic gas is homogenously mixed with the residing gas inside the test cage, we estimated the gas concentration (c) at time point (t) using the formula by Corbach [8]:

$$\frac{Cin-C}{Cin-C0}=e^{-\left(\frac{Q}{V}\right)t}$$

c_in_ concentration of the narcotic gas [%]
c_0_ concentration of the residing gas at time point t = 0 (start of the gas flow) [%]
Q flow rate of the narcotic gas [l/min]
V volume of the test cage [l].

Control animals (Air) received compressed air with a filling rate of 71% CV/min for 300 s before they were immediately sacrificed by decapitation.

#### Experimental setup

All experiments were conducted in a separate laboratory room adjunctive to the housing facilities. Mice were randomly assigned to the eight experimental groups (see 2.2.2). In order to minimize stress induced by the unfamiliar environment, all animals were habituated for 3 min on 3 consecutive days to their assigned test cage with the acrylic glass lid on top. Three days of habituation were found to be sufficient to acclimatize the animals to the new environment [55]. During this time, mice were single-housed with visible and odor contact to conspecifics. The mice were carried in their home cages to the adjunctive testing room. There, they were transferred from the home cages to their assigned test cages using individual cardboard tubes, which the animals entered voluntarily. Habituation took place from 9:00 to 11:00 a.m. and 2:00 to 4:00 p.m. The individual test cages were cleaned with paper towels between trials to retain the mouse odor simulating a home cage-like atmosphere. Control animals were additionally trained to be placed in the guillotine with the blade in a non-active position to minimize the stress prior to the decapitation procedure.

The actual experiment took place between 8:30 a. m. and 11:30 a. m. on the fourth day. After carrying the animal in its home cage to the testing room, each mouse was transferred to its assigned test cage using the individual cardboard tubes and was allowed to explore it for 1 min before the narcotic gas or air was continuously introduced. The observation period started with the beginning of gas/Air exposure and ended when the mouse reached surgical tolerance proven by the LOPR or was terminated after a maximum of 300 s. The fixed period of 300 s as cut-off time was based on previous studies, in which similar concentrations of the narcotic gases induced the LOPR after less than 300 s [8, 56]. The 300 s cut-off time was also chosen due to practical reasons as animals should be euthanized, apart from causing the least distress and suffering, within the minimal possible time.

During the induction of narcosis, behavior and vocalizations were recorded. Animals not reaching surgical tolerance within 300 s (apart from control animals) were excluded from further analysis. At the end of each experiment, animals were immediately sacrificed by decapitation within seconds and blood was collected for further analysis of glucose, adrenaline and noradrenaline concentrations. Additionally, the respiratory tract was macroscopically inspected and afterwards prepared for histological analysis (see 2.2.4). Between each trial the test cage was washed out with oxygen. Additionally, the acrylic glass lid, test cage, and movable acrylic glass board were cleaned with odorless detergent (Frosch^®^ Sensitiv Vitamin Spülmittel, parfümfrei, Erdal GmbH, Mainz, Germany) and were dried with a paper towel.

#### Assessment

**Loss of reflexes.** The righting reflex of mice is the postural response when placed on their back or side to reorient themselves such that their paws or feet are oriented towards the ground [42]. Latency to reach the LORR as an estimation of the onset of unconsciousness was recorded as it is suggested for rats [43]. Once the mouse was recumbent and immobile for 10 s, the acrylic glass board was lifted at one side by pulling at the nylon threads until the mouse rolled over to its side or back. If the mouse was unable to right itself, the animal was considered unconscious and that time point was noted [42]. In cases where the animal attempted to move or to right itself, we tested the reflex again after 10 s of immobility and repeated this procedure, if necessary. Once the righting reflex was negative, the lid of the test cage was temporarily opened after 10 s so far that the interdigital web of a hind limb could be strongly pinched with atraumatic forceps. If the mouse did not show any withdrawal movement, the animal was considered to have reached the stage of surgical tolerance. In cases of withdrawal movement of the hind limb, we tested the reflex again on the other hind limb after a further 10 s of immobility and repeated this procedure again every 15 s, if necessary. Mice were sacrificed with the onset of LOPR as at this time point it was ensured that the animals did not perceive pain during the procedure.

If a mouse reached the surgical tolerance within 300 s, the treatment was considered practicable. We defined the treatment as reliable if 15 out of 16 (93.8%) mice within one group reached the surgical tolerance in ≤ 300 s. This definition is an approximation to the AD_95_, i. e. the anesthetic dose at which 95% of the patients are anesthetized and will not show any movement to pain stimuli [57]. In our study, experimental groups with a minimum of 14 out of 16 animals (i. e. 87.5%) reaching surgical tolerance within 300 s were included in the statistical analysis to demonstrate a dose-response dependency. If a single mouse did not reach the surgical tolerance in ≤ 300 s, the animal was excluded from further analysis.

**Behavioral analysis.** The behavior from the start of gas exposure to the end of the experiment (either reaching LOPR or after a maximum of 300 s) was videotaped and afterwards analyzed by an experimenter blind to the treatment. Since it is generally suggested that animals can only perceive pain, suffering, and distress during stage 1 of anesthesia, we observed which of the following behavioral parameters occurred before the LORR:

**Locomotion**. Crossings (i. e. with the middle part of the mouse body) of a virtual center line parallel to the short side of the test cage according to Niel and Weary [58]. The number [n] of crossings was counted and calculated per min. As we wanted to measure ambulatory behavior, we excluded incomplete crossings in form of body turns which mostly occurred within 3 s. Increased locomotor activity can be a sign of agitation and distress. In addition, we looked out for freezing behavior as a sign of fear and anxiety.
**Rearings**. The number [n] of rearings (i. e. lifting both fore paws) was recorded and calculated per min.
**Stress-induced grooming**. Stress-induced grooming behavior was defined as ‘‘incorrect” transitions between different grooming stages (uninterrupted cephalocaudal progression of self-grooming) and as more interrupted grooming bouts [59]. The number [n] of incorrect grooming actions was noted and calculated per min.
**Jumps**. Since CO_2_ exposure can induce escape responses, such as jumping behavior, in mice [51], we additionally counted the number of jumps (defined as sudden springing with all four paws off of the ground) per min.
**Excitatory phenomena**. Clonic or tonic convulsions may be evoked especially during slow induction or emergence of anesthesia. Hence, the occurrence of the following excitatory phenomena according to Irwin [44] were recorded and calculated as a percentage for each group: running excitement, clonus (coordinated, asymmetrical convulsion with natural, purposeful-like movements, e. g. "running", while the animal is lying down or on its side), and opisthotonus (seizure where head, body, and limbs are rigidly extended and arched backwards) [44].
**Gait**. The occurrence of the following movements were registered and the percentage for each group calculated according to Irwin [44]: ataxia (inability of truncal, pelvic, and limb muscles to move in unison, so that the animal tends to excessively sway, rock, or lurch to the side as it proceeds forward and is variously unable to walk a straight line) and hypotonic gait (impairment due to limb weakness or paralysis in which the animal is variously unable to support its weight but can proceed forward in a straight line without lurching).
**Vocalization**. The frequency and duration of audible and ultrasound calls were recorded by the Avisoft-RECORDER 2.7 and analyzed by the Avisoft-SASLab Pro 4.15 software (Avisoft Bioacoustics, Glienicke, Germany).

**Blood analysis** At the end of the experiment, the animals were immediately decapitated and trunk blood was collected (dripped directly or drawn up with a 1 ml syringe) for further analysis.

**Blood glucose concentration**. Glucose concentration [mmol/l] in the trunk blood was immediately determined using a blood glucose meter by Ascensia^®^ Dex2^®^ with a measuring range of 10–600 mg/dl (0,6–33,3 mmol/l) and Autodisc® test strips (both Bayer HealthCare AG, Leverkusen, Germany) according to the manufacturer’s instructions.
**Plasma adrenaline and noradrenaline concentrations**. Trunk blood was dripped into ice cooled tubes (420 A weiß Katecholamine 1 ml containing EGTA/GSH, Kabe Labortechnik, Nümbrecht-Elsenroth, Germany) and immediately centrifuged (5°C, 10 min, 4000 rpm, Eppendorf Centrifuge 5403, Hamburg, Germany). The supernatant plasma was collected and stored in another tube in the fridge (-12 to -14°C) until further processing on the same day using the test kit by CHROMSYSTEMS GmbH for analyzing catecholamine concentrations (Munich, Germany). The test kit consisted of a mobile phase, internal standard, extraction buffer, washing buffer, elution buffer, sample clean up columns, plasma calibration standard for catecholamines and plasma endocrine control, pathological range, lyophilized. Plasma samples were less than 1 ml. Therefore, we modified the manufacturer’s instruction as following: the plasma was diluted with 0.9% sodium chloride (NaCl) solution and the internal standard was adjusted quantitatively as preliminary tests have already revealed substantial differences in catecholamine plasma concentrations between CO_2_ and the halogenated ethers (treatment groups CO_2_ 20, CO_2_ 60, and CO_2_ 100: 100 μl plasma, 800 μl 0.9% NaCl, and 100 μl internal standard, treatment groups Iso 2%, Iso 5%, Sevo 4.8%, and Sevo 8% and control group: 200 μl plasma, 725 μl 0.9% NaCl, and 75 μl internal standard). The probes were stored at -80°C until they were analyzed for catecholamine concentrations by high pressure liquid chromatography (HPLC). HPLC analysis was conducted with electrochemical detection (pump 510, autosampler 717 and detector 460 by Waters, Milford, Massachusetts, USA; with 0.8 ml/min at 400 mV) using an equilibrated and tested column for catecholamines in plasma and the mobile phase for HPLC analysis of catecholamines by CHROMSYSTEMS GmbH (Munich, Germany) and the chromatography software Peak Net^TM^ (automatization software, version 5.1, Dionex, Sunnyvale, California, USA). The concentrations of the catecholamines in plasma samples were calculated relative to the internal standard. Each run was 20 min, and the retention times for adrenaline and noradrenaline were 6.0 min and 7.1 min, respectively. The plasma concentrations of adrenaline and noradrenaline are expressed as [ng/ml].

Examination of the respiratory tract.

Gross and histological alterations of the respiratory tract were taken as indications of pain.

**Gross examination**. Nasal and buccal mucosae of the respiratory tract as well as the conjunctivae were examined for mucosal defects and signs of inflammation (redness, swelling, and exudation). Moreover, it was noted if there was foamy efflux from the trachea of the decapitated trunk. As hemorrhage has been observed following CO_2_ euthanasia in rats [24], the tracheal lumen was controlled for coagulated blood caused by intrapulmonary bleeding.

**Histological examination**. Both lungs including the trachea were carefully dissected and fixed by immersion in 4% paraformaldehyde for 14 days. Afterwards, the specimens were completely embedded in Paraplast^®^ (Roth, Karlsruhe, Germany) and 6 μm slices were prepared. Slices were stained with hematoxylin and eosin (HE), connective tissue staining (Ladewig), and Elastika staining (resorcin fuchsine, thiazine red, picric acid). A horizontal plane was chosen as section plane that included parts of the trachea, bronchi, bronchioles, and alveoli. Lung slices of 4 randomly selected mice per group were scanned by a blinded investigator. Per animal, 2 HE, 1 Ladewig, and 1 Elastika stained slices with 200 x and 400 x magnification of the microscope (Axioskop HBO 50, Carl Zeiss AG, Oberkochen, Germany; microscope camera: Nikon DS Ri 1, Nikon Instruments Europe B.V., Amstelveen, Netherlands) were analyzed for the occurrence of blood aspiration in trachea and bronchi, congestion of blood vessels (veins, venules, and capillaries), and alteration to lung tissues (bleeding into alveolar septa and alveolar spaces, atelectasis, edema, and disintegration).

#### Statistical analysis

All data were analyzed using SigmaPlot^®^ (Version 11, SPSS Inc., Chicago, Illinois, USA) and are shown as medians and [25./75. percentile] or percentage [%] of animals of each experimental group. Only mice that reached the surgical tolerance within 300 s and experimental groups in which a minimum of 14 mice (87.5%) reached the surgical tolerance within 300 s as well as all animals of the control groups were included in the final analysis (see Table 1, grey shaded groups). The majority of data was not normally distributed, therefore all data were analyzed by Kruskal-Wallis one-way ANOVAs on ranks followed by Dunn’s tests. The behavioral data as well as glucose, adrenaline, and noradrenaline concentrations were compared to control (Air) and also pairwise between the treatment groups. Data of LORR and LOPR were only compared pairwise between the treatment groups. A probability value of *p*<0.05 was considered to be statistically significant. The treatment effects on the respiratory tract (gross and histological examination) were descriptively analyzed.

**Table 1:** Latencies [s] to reach the loss of righting reflex (LORR) and pedal withdrawal reflex (LOPR) are presented as median [25./75. percentiles] of mice that reached the LOPR within 300 s.

**NMRI**	**LORR [s]**		**LOPR [s]**		**LOPR [n / %]**
CO_2_ 20	111.5	[108.0/114.0]	261.0	[197.5/298.5]	4 / 25
CO_2_ 60	58.5	[55.0/66.5]	94.5	[74.5/129.0]	16 / 100
CO_2_ 100	51.0	[46.75/54.75]	65.0	[60.5/71.25]*	15 / 93.8
Iso 2%	191.5	[181.0/204.0]	284.0	[256.0/297.0]	10 / 62.5
Iso 5%	80.5	[73.0/86.0]*^+^	101.5	[95.0/124.0]^+^	16 / 100
Sevo 4.8%	169.0	[141.0/181.0]	270.5	[246.0/295.0]	10 / 62.5
Sevo 8%	100.5	[86.0/113.0]*^+^	141.0	[127.0/172.0]*^+^	14 / 87.5
**C57Bl/6J**	**LORR [s]**		**LOPR [s]**		**LOPR [n / %]**
CO_2_ 20	108.0	[84.5/117.5]	196.0	[166.0/267.25]	5 / 31.3
CO_2_ 60	60.0	[57.0/63.75]	80.0	[75.25/85.75]	15 / 93.8
CO_2_ 100	50.0	[49.0/52.5]	63.5	[60.5/67.0]	16 / 100
Iso 2%	122.0	[108.0/136.0]*^+^	250.5	[231.0/282.0]*^+^	14 / 87.5
Iso 5%	67.0	[65.5/68.0]^+^^#^	95.0	[83.5/102.0]^+^^#^	16 / 100
Sevo 4.8%	122.0	[113.25/129.5]	230.0	[213.5/259.5]	13 / 81.3
Sevo 8%	82.0	[75.5/86.0]*^+^	115.5	[109.0/123.0]*^+^	16 / 100

Note, the latencies to reach the LOPR have to interpreted with caution as the opening of the cage lid to test this reflex might have diluted the gas concentration with air. The number [n] and percentage [%] of a group reaching the LOPR within 300 s are indicated in the right column. Grey boxes indicate the groups that are included in the statistical analysis of behavioral and blood parameters as well as the descriptive analysis of the respiratory tract. p<0.05 (Dunn´s test),

* vs. CO_2_ 60,

^+^ vs. CO_2_ 100,

^#^ vs. Iso 2%.

### Experiment 2

The approach-avoidance test was conducted according to Makowska et al. [52]. The principle of this test is that animals are put in a situation where they have to decide between the intake of a food reward or the escape from a negative stimulus, in this case incoming inhalant gas. First, mice are trained to enter the experimental cage on a signal, which is rewarded with food. Once the training is accomplished, the animals are exposed in the experimental cage to the inhalant gas, which was turned on when the animal starts eating. The latency of the animal to leave the cage is recorded. Aversion is defined as significantly decreased escape latency after gas exposure compared to Air control. In the present study, we used 16 male (30.9 ± 2.9 g) and 14 female (22.6 ± 1.53 g) C57Bl/6J mice. The animals were randomly allocated to the three treatment groups, i. e. isoflurane (4 female/6 male), sevoflurane (5 female/5 male), and CO_2_ (5 female/5 male). All treatment groups were initially exposed to air and then to one of the three gases in three ascending concentrations, so that each animal served as its own control.

#### Apparatus

**Fig 2:**
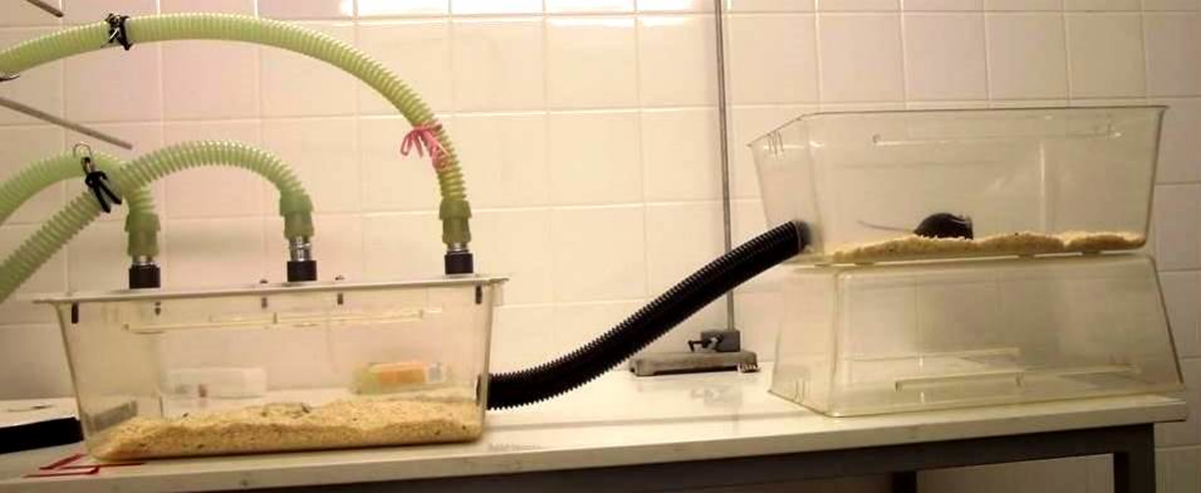
Set-up of the approach-avoidance test. The lid of the left chamber was the same as the one used for *Experiment 1*.

The apparatus consisted of two Macrolon type III cages (420 x 265 x 150 mm, floor area 825 cm^2^, Bioscape, Wandlitz, Germany) connected with a black, ribbed hose (Ø 3.5 cm, 35 cm long) that allowed the animal to cross freely between both cages. The experimental cage was placed 15 cm below the other cage (see Fig 2). Both cages contained normal bedding material and provided access to standard diet and tap water. Environmental enrichment (nesting material, cardboard tube) was only provided in the upper cage. For training and testing, the standard cage lid of the experimental cage was substituted with an acrylic glass lid designed for Experiment 1 (see. 2.2.1). During the whole experiment, the animals were single-housed in the testing apparatus with visible and odor contact with conspecifics.

#### Experimental setup

**Fig 3:**
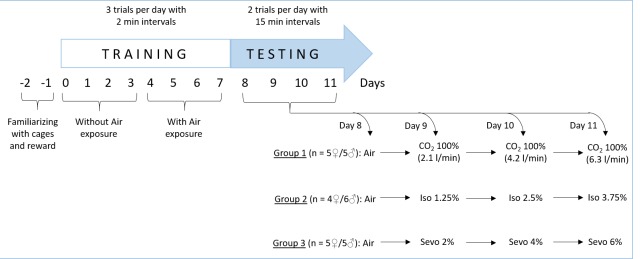
Overview of the timeline of the approach-avoidance test.

An overview of the timeline for the training and testing procedure is provided in Fig 3.

**Training.** Two days before the actual training started, animals were familiarized with the two cages, tube, and reward. It was ensured that the animals entered both cages via the connecting tube. Additionally, the reward consisting of sweetened condensed milk (Milchmädchen, Nestlé, Frankfurt, Germany, plus gelatin (1:1)) was presented once before training in the upper cage. The actual training took place on the following eight days. The aim of training was to condition the animal to enter the lower experimental cage on a signal, i. e. by knocking with a fingernail on the cage wall, which was rewarded with food. During the training phase, the normal lid of the experimental cage was removed and substituted by the experimental acrylic glass lid. Firstly, the animal was allowed to explore both cages for 120 s. Then, if the animal did not voluntarily enter the upper cage, it was placed there by the experimenter and the tube was closed with a rubber plug. Afterwards, the reward was placed in the lower experimental cage and the tube was re-opened. Once the animal stepped downwards, it was accompanied with the signal. For 240 s, the animal was allowed to stay in the experimental cage and to eat the reward. This procedure was repeated three times per day with 2-min intervals on four consecutive days. Over the next four days, the animal was trained to enter the experimental cage that had an incoming airflow to acclimate them to the air movement and noise. On the first day, the airflow (4 l/min) was running on before the animal entered the experimental cage. On the following three days, the airflow (in ascending strength 4, 7, and 10 l/min) started once the animal entered the experimental cage and commenced eating. This procedure was also repeated three times per day with 2-min intervals.

**Testing.** Testing took place on four consecutive days. On the first testing day, each animal was exposed to air (10 l/min) as control. On the following three testing days, the animal received one of the three gases in ascending concentrations. The concentrations were chosen based on the findings of Experiment 1. We used 25%, 50%, and 75% of the lowest concentration that reliably induced the LOPR (see Table 1). Hence, isoflurane and sevoflurane were introduced with a flow rate of 10 l/min in concentrations of 1.25, 2.5, and 3.75% (isoflurane) and 2, 4, and 6% (sevoflurane). CO_2_ was tested with flow rates of 2.1, 4.2, and 6.3 l/min at a concentration of 100%. On each testing day, the animal was allowed to explore both cages for 120 s. Afterwards the mouse was locked in the upper cage for 60 s. After removing the plug, the mouse was guided by the signal into the experimental cage. Once the animal started eating the reward, the gas was immediately turned on and the time latencies to leave the experimental cage (with all four paws in the tube) were recorded. The trial was finished when the animal left the cage or was ended after 240 s. For each gas and concentration, two test trials per day were conducted as replicates with a 15-min interval. During the resting time, the experimental cage was ventilated with air to avoid gas contamination from the first trial, and bedding material was changed. In order to avoid extinction from the conditioning, an additional training trial with air (10 l/min) was conducted in the afternoon.

#### Statistical analysis

Two female animals had to be excluded from the statistical analysis due to insufficient training: one from the CO_2_ group and one from the sevoflurane group. The mean of the two trials per day was calculated for each mouse and gas concentration. If an animal lost consciousness during one trial, both trials of the animal were excluded from further statistical analysis (one animal for the lowest and three animals for the highest isoflurane concentration; two animals for the highest sevoflurane concentration). Afterwards the means of each treatment group and concentration were generated. A two-way ANOVA on repeated measures with factor treatment (CO_2_, Iso, Sevo) and factor gas concentration (0% (=Air), 25%, 50%, 75%) was conducted followed by Holm-Sidak test versus control (Air). As it has been shown that previous exposure can influence the aversion of a given gas [22], we analyzed the first gas exposure, i. e. 25%, separately using a one-way ANOVA. A probability value of *p*<0.05 was considered to be statistically significant.

## Results

### Loss of reflexes, practicability, and reliability

Latencies of the onset of LORR (i. e. unconsciousness) and LOPR (i. e. state of surgical tolerance) are shown in Table 1 as medians and [25./75. percentile]. Note that the absolute figures for LOPR have to be interpreted with caution as the opening of the cage lid to test this reflex likely caused a slight dilution of the anesthetic gas with ambient air and, hence, might have led to a delayed onset of this reflex. Despite that, LOPR was, independent from the strain, most rapidly reached when animals were treated with CO_2_ 100, followed by CO_2_ 60 and Iso 5%. According to our definitions in 2.2.4, CO_2_ 60, CO_2_ 100, and Iso 5% can be seen as practical and they reliably induced the stage of surgical tolerance within 300 s (i. e. in ≥ 93.8% of the group) in both mouse strains. This was also true for using Sevo 8% in C57Bl/6J mice, whereas for NMRI mice, two animals did not reach surgical tolerance within 300 s. In both strains, the percentage of animals reaching surgical tolerance following CO_2_ 20, Iso 2%, and Sevo 4.8% exposure was below 93.8% and therefore, can be seen as impracticable and unreliable according to our definition (Table 1). In approximation to the AD_95_, experimental groups with minimum of 14 out of 16 (87.5%) effective treatments were also included in the further statistical analysis to demonstrate a dose-response dependency.

In NMRI mice, we detected significant differences regarding unconsciousness (H = 50.7, p<0.001, Df = 3): The latencies of LORR for CO_2_ 100 and CO_2_ 60 were significantly shorter than those for Iso 5% and Sevo 8%, respectively. LOPR was most rapidly reached after CO_2_ 100 exposure compared to CO_2_ 60, Iso 5% as well as to Sevo 8%. In addition, the latency for LOPR for CO_2_ 60 was significantly shorter than Sevo 8% (H = 49.3, p<0.001, Df = 3) (see Table 1).

In C57Bl/6J mice, the latencies for LORR (H = 66.7, p<0.001, Df = 4) and LOPR (H = 66.4, p<0.001, Df = 4) of animals treated with CO_2_ 100 were shorter than for animals treated with Iso 2%, Iso 5%, and Sevo 8%. Also, CO_2_ 60 induced the LORR and LOPR faster than Iso 2% and Sevo 8%. LORR and LOPR latencies of Iso 2% were also significantly prolonged when compared to Iso 5% (see Table 1).

The estimated concentrations of the gases in the test cage were calculated for the onset of the LORR and the LOPR for each narcotic gas and strain and are shown in Table 2.

**Table 2:** The estimated concentrations in percent [%] of the chamber volume (CV) for all analyzed narcotic gases and both mouse strains at the onset of LORR and LOPR.

NMRI	Gas concentration [%] CV	
	LORR	LOPR^1)^
CO_2_ 20	31.1	58.1
CO_2_ 60	44.3	61.0
CO_2_ 100	57.3	66.2
Iso 2%	1.8	1.9
Iso 5%	3.1	3.5
Sevo 4.8%	4.2	4.6
Sevo 8%	5.6	6.5
**C57Bl/6J**	**Gas concentration [%] CV**	
	**LORR**	**LOPR**^**1)**^
CO_2_ 20	30.3	48.0
CO_2_ 60	45.1	55.1
CO_2_ 100	56.6	65.3
Iso 2%	1.5	1.9
Iso 5%	2.7	3.4
Sevo 4.8%	3.7	4.5
Sevo 8%	5.0	6.0

^1)^ Please note that for LOPR this is a rough calculation due to anesthetic gas dilution with ambient air.

### Behavioral analysis

Table 3 provides an overview of the results for the behavioral parameters. All behaviors were observed before the onset of LORR and therefore changes could be a sign of distress. Significant changes (up- and down-regulation) in comparison to Air control are indicated by arrows.

**Table 3:** Significant changes in behavioral parameters in comparison to air control are indicated by arrows either as up- or down-regulation or as no changes (—).

NMRI	CO_2_ 60	CO_2_ 100	Iso 5%	Sevo 8%
Locomotion	**—**	**—**	**⇧**	**⇧**	
Rearings	**⇩**	**⇩**	**—**	**—**	
Stress-induced grooming	**—**	**—**	**—**	**—**	
Clonus	**—**	**—**	**⇧**	**⇧**	
Running excitement	**—**	**—**	**⇧**	**⇧**	
Opisthotonus	**—**	**—**	**⇧**	**⇧**	
Jumps	**—**	**—**	**—**	**—**	
Ataxia	**⇧**	**⇧**	**⇧**	**⇧**	
Hypotonic gait	**⇧**	**⇧**	**—**	**—**	
**C57Bl/6J**	**CO**_**2**_ **60**	**CO**_**2**_ **100**	**Iso 2%**	**Iso 5%**	**Sevo 8%**
Locomotion	**—**	**—**	**—**	**⇧**	**⇧**
Rearings	**⇩**	**⇩**	**—**	**—**	**—**
Stress-induced grooming	**—**	**—**	**⇩**	**—**	**—**
Clonus	**—**	**—**	**—**	**—**	**⇧**
Running excitement	**—**	**—**	**⇧**	**⇧**	**⇧**
Opisthotonus	**—**	**—**	**—**	**—**	**—**
Jumps	**—**	**—**	**—**	**—**	**—**
Ataxia	**⇧**	**⇧**	**⇧**	**⇧**	**⇧**
Hypotonic gait	**⇧**	**⇧**	**—**	**—**	**—**

#### Locomotion

In NMRI mice, there was a significant treatment effect on locomotion (H = 44.7, p<0.001, Df = 4): groups treated with Iso 5% (10.2 [7.7/12.8]) and Sevo 8% (8.6 [7.6/10.4]) showed significantly more ambulatory behavior per min than control animals (3.7 [2.9/4.6]). Animals treated with Iso 5% were more active than animals treated with CO_2_ 60 (4.9 [3.4/6.0]) and CO_2_ 100 (6.00 [4.3/8.8]). In addition, Sevo 8% induced more locomotor activity than CO_2_ 60.

A similar treatment effect was observed for C57Bl/6J mice (H = 41.7, p<0.001, Df = 5): locomotion was significantly higher in groups treated with Iso 5% (5.3 [3.8/6.2]) and Sevo 8% (6.7 [4.3/7.5] compared to control animals (1.8 [1.2/2.5]). Iso 5% treated C57Bl/6J mice were more active than when exposed to CO_2_ 100 (2.9 [1.6/3.8]). The highest locomotor activity was observed for mice exposed to Sevo 8% when compared to CO_2_ 60 (3.5 [2.3/4.1]) and CO_2_ 100. Freezing behavior did not occur in any group.

#### Rearings

Effects on rearings were mainly registered for animals treated with CO_2_. In NMRI mice, animals exposed to CO_2_ 60 (3.6 [1.5/9.0]) and CO_2_ 100 (7.1 [3.4/7.8]) showed significantly fewer rearings per minute (H = 20.4, p<0.001, Df = 4) compared to control animals (10.6 [7.9/12.2]) and animals exposed to Iso 5% (10.9 [7.2/17.2]).

Also in C57Bl/6J mice, CO_2_ 60 (1.3 [0.0/1.4]) and CO_2_ 100 (1.4 [0.0/1.9]) induced significantly fewer rearings per minute (H = 45.3, p<0.001, Df = 5) compared to control animals (5.9 [4.2/7.0]) as well as compared to animals exposed to Iso 5% (4.3 [3.2/7.3]). For CO_2_ 60, the number per minute for rearings was significantly less than for Iso 2% (2.9 [2.3/5.7]).

#### Stress-induced grooming

Stress-induced self-grooming was rarely observed and was mainly registered in control animals: NMRI mice displayed 0.5 [0.2/0.6] and C57Bl/6J 0.6 [0.4/0.6] stress-induced grooming events per minute. Stress-induced grooming was also registered in NMRI mice exposed to Iso 5% (0.0 [0.0/0.8]) and in C57Bl/6J mice exposed to Iso 2% (0.0 [0.0/0.5]), however, to a lesser amount than in control mice (H = 51.3, p<0.001, Df = 5).

#### Jumps

Escape-oriented jumping was a rare event, mainly in NMRI mice: animals exposed to CO_2_ 100 showed 0.0 [0.0/3.2] jumps per minute, 0.0 [0.0/0.5] when exposed to Iso 5%, and 0.0 [0.0/0.8] when exposed to Sevo 8%. In C57Bl/6J, some jumps per minute (0.0 [0.0/0.8]) were registered in animals exposed to CO_2_ 60. Although one-way ANOVA on ranks revealed a significant treatment effect for both strains (NMRI: H = 9.7, p = 0.046, Df = 4; C57Bl/6J: H = 11.5, p = 0.042, Df = 5), post-hoc analysis failed to detect significant differences.

#### Excitatory phenomena

No excitatory phenomena like clonus, running excitement, and opisthotonus were observed for both mouse strains when exposed to CO_2_ 60, CO_2_ 100, and Air. The three excitatory events only occurred when animals were exposed to the two halogenated ethers. In NMRI mice, all animals (100%) treated with Iso 5% and Sevo 8% showed clonus and running excitement. Clonus only occurred in 25% of C57Bl/6J mice exposed to Sevo 8%. Running excitement was registered in 57% of C57Bl/6J mice exposed to Iso 2%, in 75% of the Iso 5% group and in 100% of the Sevo 8% group. Considerable strain differences were seen with the phenomenon opisthotonus: it was observed in 25% of NMRI mice exposed to Iso 5% and in 64% of animals exposed to Sevo 8%, whereas it did not occur at all in C57Bl/6J mice.

#### Gait

All Air control animals of both strains (NMRI and C57Bl/6J) did not show any signs of ataxia or hypotonic gait. In both strains, hypotonic gait only occurred when animals were exposed to to CO_2_ 60 or to CO_2_ 100. Ataxia occurred to all animals independent from the strain or the inhalant gases in different concentrations.

#### Vocalization

In both strains, we could not detect any vocalizations (audible and ultrasound) in control animals (Air) or in treatment groups.

### Blood parameters

#### Blood glucose concentration

**Fig 4:**
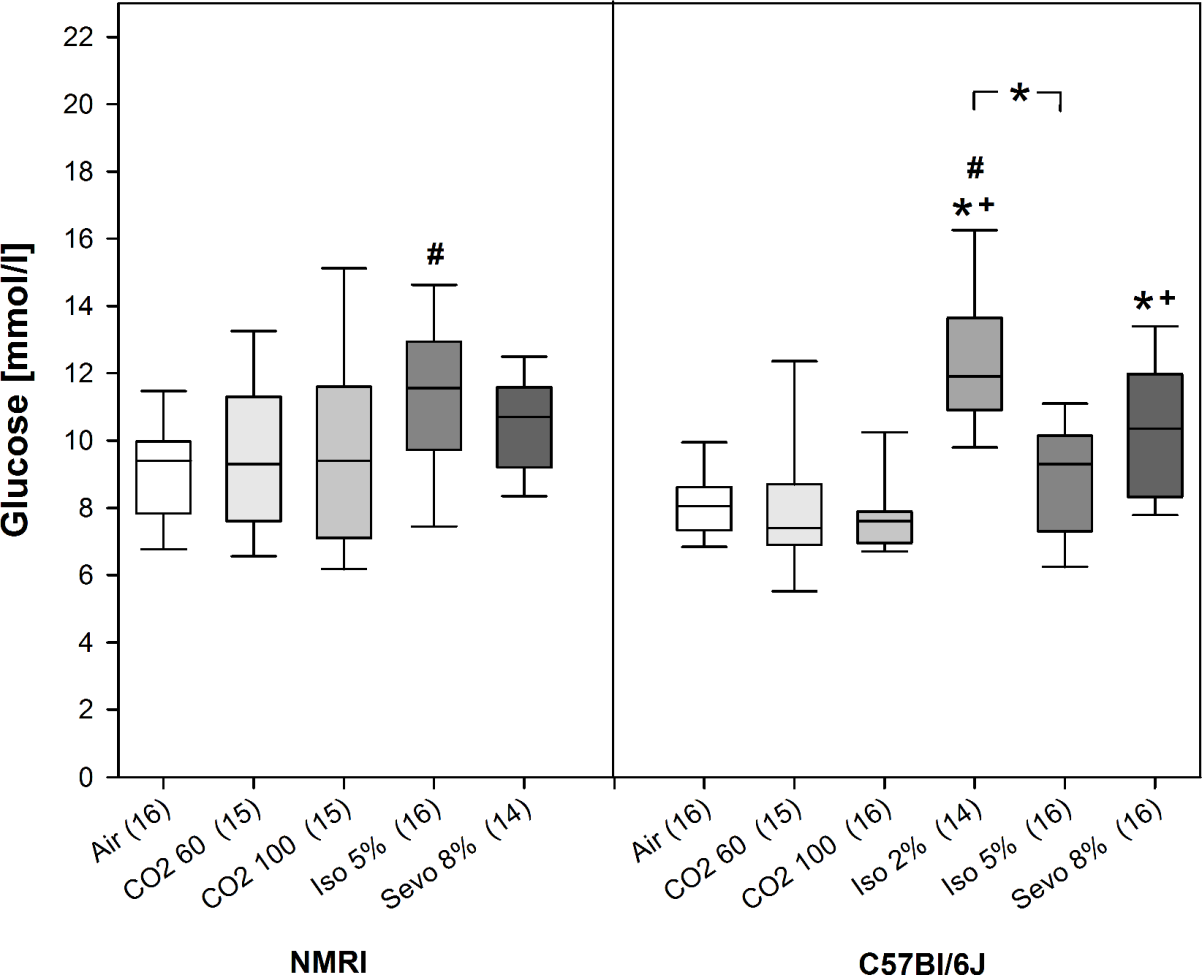
Blood glucose levels [mmol/l] for air control and animals exposed to the three inhalant gases at the beginning of surgical tolerance as median [25./75. percentiles]. Number of mice per treatment group in brackets. * p<0.05, beginning and end of the bar point to the groups compared.

In NMRI mice, we could not detect a difference in the glucose concentrations between the three treatment groups. However, glucose concentration of animals exposed to Iso 5% was significantly increased compared to Air control animals (H = 10.3, p = 0.037, Df = 4; see Fig 4). In C57Bl/6 mice, blood glucose concentrations significantly differed between treatment groups (H = 39.4, p<0.001, Df = 5): the highest concentration was found when mice were exposed to Iso 2% in comparison to CO_2_ 60, CO_2_ 100, Iso 5%, and also to Air control. Additionally, glucose concentrations of animals exposed to Sevo 8% were significantly higher compared to CO_2_ 60 and CO_2_ 100 (see Fig 4).

#### Plasma adrenaline and noradrenaline concentrations

**Fig 5:**
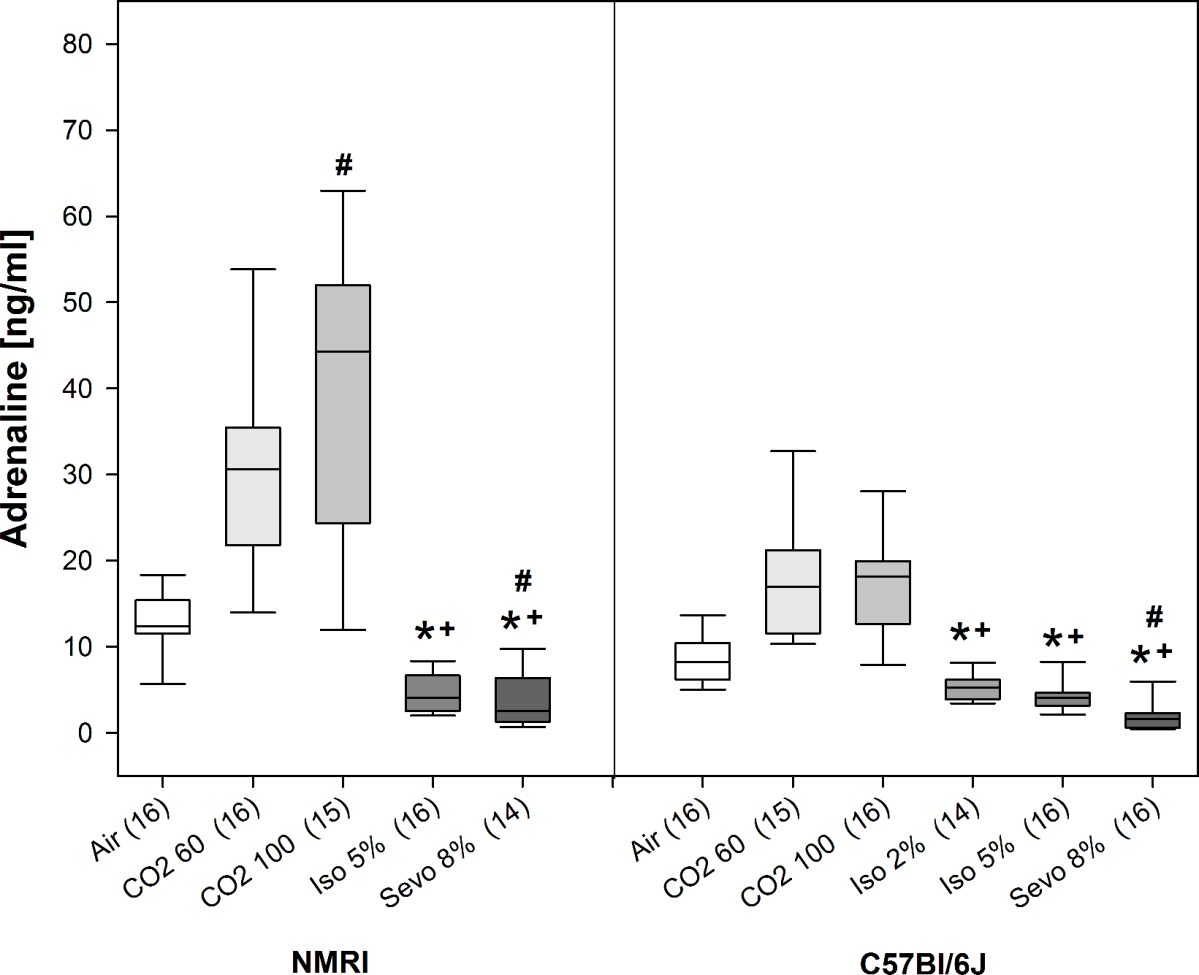
Plasma adrenaline levels [ng/ml] for air control and animals exposed to the three inhalant gases at the beginning of surgical tolerance as median [25./75. percentiles]. Number of mice per treatment group in brackets. * p<0.05, beginning and end of the bar point to the groups compared.

Significant treatment effects on plasma adrenaline concentrations were observed for both NMRI (H = 59.2, p<0.001, Df = 4) and C57Bl/6J mice (H = 73.0, p<0.001, Df = 5) (see Fig 5). Post-hoc analyses revealed that for both strains, adrenaline concentrations of animals exposed to CO_2_ 60 and CO_2_ 100 were significantly increased compared to isoflurane and sevoflurane in all concentrations. Adrenaline concentration of CO_2_ 100 treated NMRI mice was significantly higher than in Air control animals. In C57Bl/6J mice, CO_2_ exposure also seemed to increase adrenaline concentrations in comparison to Air control, although post-hoc analysis did not reveal significance. Interestingly, after Sevo 8% exposure adrenaline concentrations were decreased in comparison to Air control in both strains (see Fig 5).

**Fig 6:**
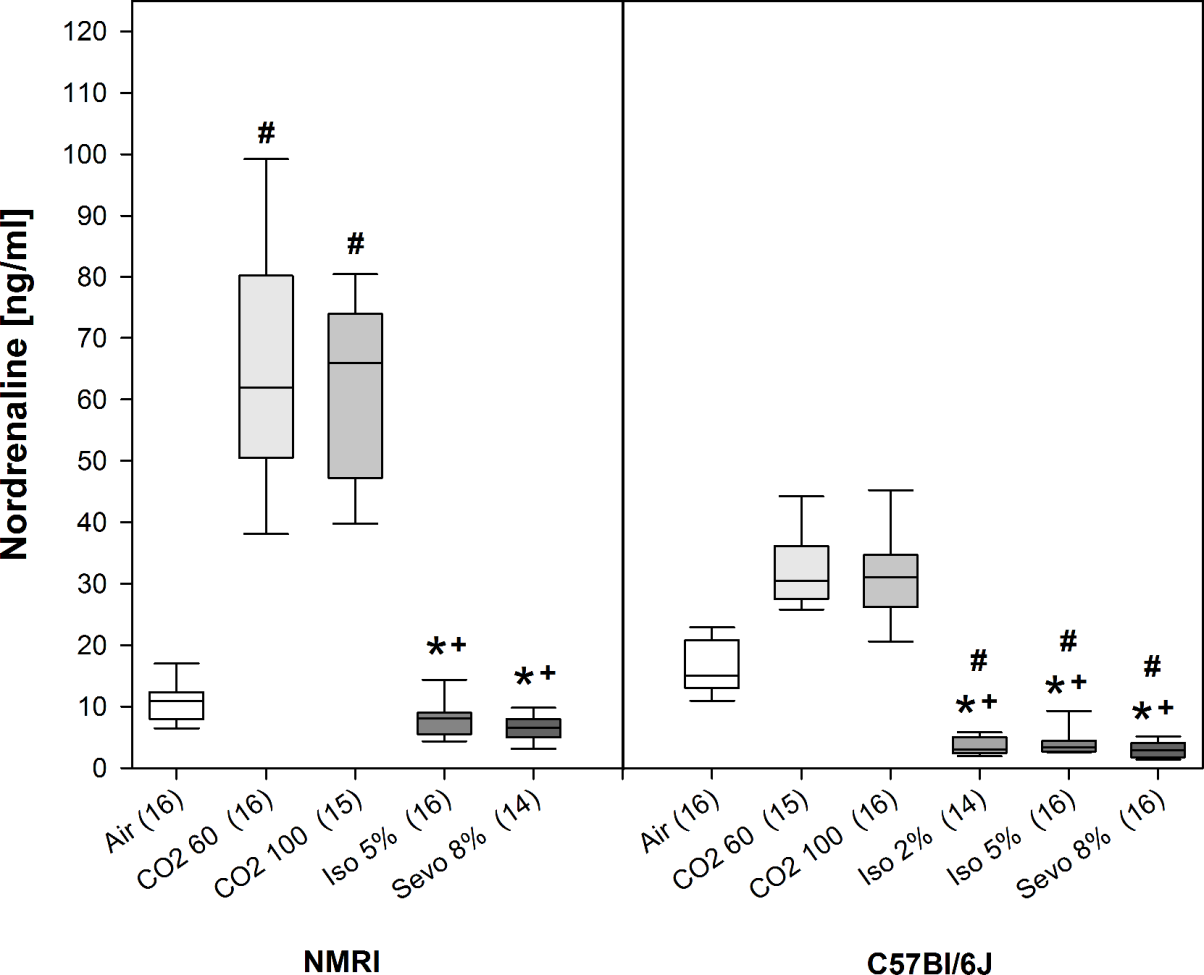
Plasma noradrenaline levels [ng/ml] for air control and animals exposed to inhalant gases at the beginning of surgical tolerance as median [25./75. percentiles]. Number of mice per treatment group in brackets. * p<0.05, beginning and end of the bar point to the groups compared.

We observed a similar treatment effect on the plasma noradrenaline concentrations for NMRI (H = 59.1, p<0.001, Df = 4) and C57Bl/6J mice (H = 76.8, p<0.001, Df = 5) (see Fig 6). In NMRI mice, noradrenaline concentrations were increased in animals exposed to CO_2_ 60 and CO_2_ 100 in comparison to Iso 5% and Sevo 8% and, additionally, compared to Air control. In C57Bl/6J mice, CO_2_ 60 and CO_2_ 100 also showed higher noradrenaline concentrations compared to isoflurane and sevoflurane in all concentrations, however, this effect was not significant when compared to Air control. Also for noradrenaline, we observed significant lower concentrations for animals exposed to Iso 2%, Iso 5% and Sevo 8% compared to Air control (see Fig 6). For all animals, we did not observe any recovery or movement after being removed from the narcotic chamber which could have influenced the catecholamine concentrations.

### Respiratory tract

#### Gross examination

In both strains, gross examination of mucosae, trachea and lung revealed no mucosal alterations or signs of inflammation (redness, swelling, and exudate) in control animals or in treatment groups. In NMRI mice, foamy efflux from trachea of the decapitated trunk was noticed in several animals of all treatment groups (CO_2_ 60 (5 animals), CO_2_ 100 (2), Iso 5% (10), Sevo 8% (4)), but it did not occur in control animals.

In C57Bl/6J mice, foamy efflux was noted in some animals from every group except in group CO_2_ 100 (control (1 animal), CO_2_ 60 (1), Iso 2% (6), Iso 5% (7), Sevo 8% (7)). In both strains, we observed coagulated blood in the tracheal lumen induced by decapitation in several animals (3–12) of all treatment as well as control groups.

#### Histological examination

In both strains and in all groups (control and treatment), we found massive numbers of erythrocytes and other blood cells in trachea and/or bronchi. For all groups, congestion of blood vessels (veins, venules, and capillaries), bleeding into alveolar septa and alveolar spaces could not always be differentiated from each other and can be rather considered as transitional stages of lung hemorrhage. Disintegration, i. e. loss of the typical order of alveolar septa and spaces, of lung tissue was observed in several NMRI mice treated with CO_2_ 60 and Sevo 8%. We could not detect edema in any of the examined animals. We diagnosed atelectasis in both strains in mice exposed to CO_2_ 60 and 100.

### Experiment 2

**Fig 7:**
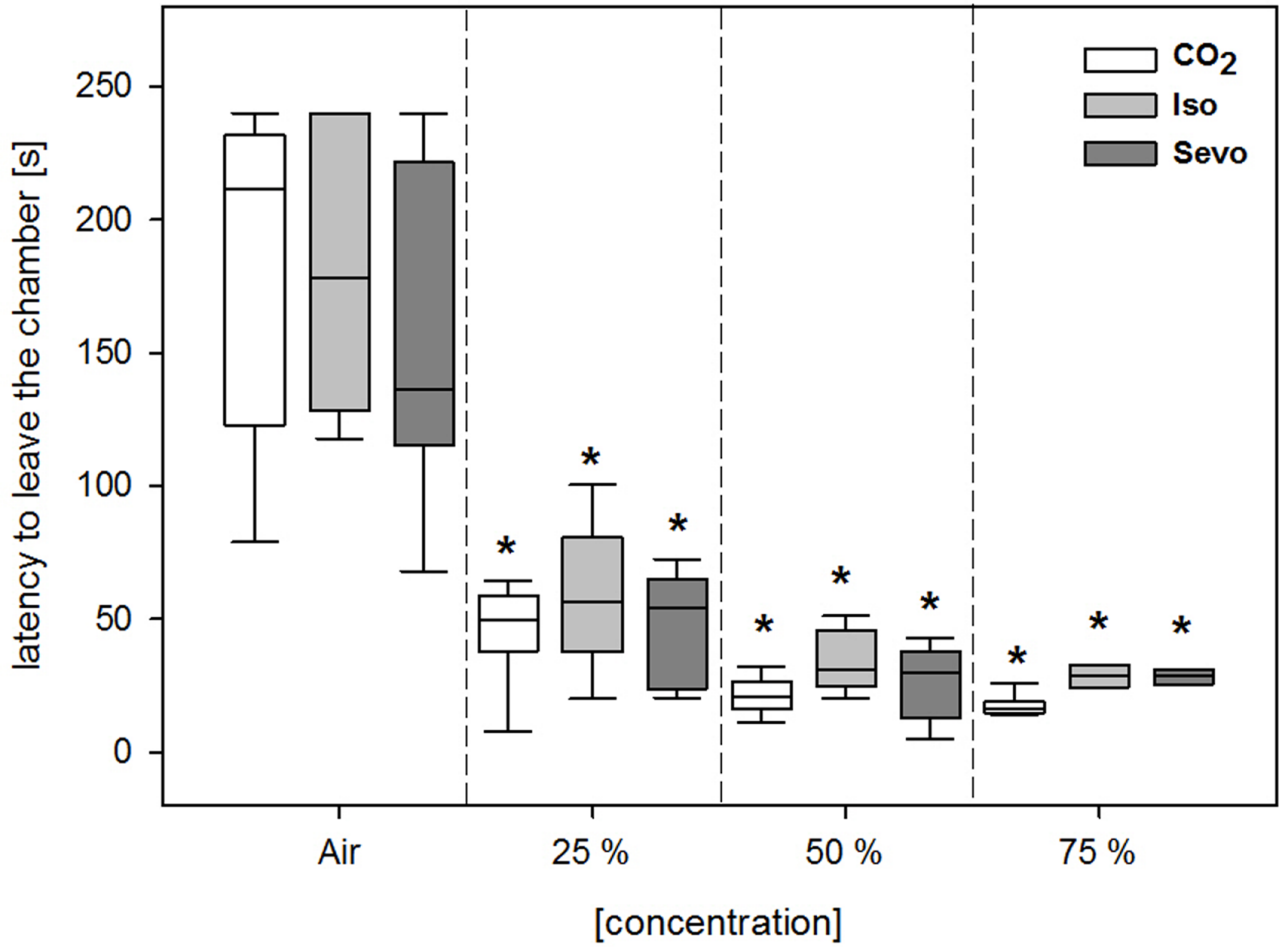
Escape latencies [s] from the narcotic chamber of the approach-avoidance test after the exposure to air and different concentrations of CO_2_, isoflurane (Iso), and sevoflurane (Sevo). Data are shown as median [25./75. percentiles]. * p<0.05 vs. Air control.

Independent from the concentration, all three gases were perceived similar aversive by the animals, indicated by shorter escape latencies compared to Air control (F(3, 69) = 147.95; p<0.001; see Fig 7) and no significant treatment effect (F(2, 69) = 1.83; p = 0.18). The separate analysis of the first gas exposure also revealed no significant difference between the three inhalant gases (p = 0.473). However, it has to be noted that 4 animals treated with isoflurane (i. e. 1 animal for the lowest and 3 animals for the highest isoflurane concentration) and 2 animals treated with the highest sevoflurane concentration had to be taken out of the apparatus preliminary as they became recumbent during the gas exposure.

## Discussion

Within the scope of refinement, the primary goal of our study was to identify the least severe of three inhalant gases, i. e. isoflurane, sevoflurane, and CO_2_, to induce the loss of consciousness in order to kill laboratory mice in the most humane manner. When assessing impairment of animal welfare in conjunction with general anesthetics, the spectrum of adverse drug effects, their severity, and duration of severity have to be taken into account. In this regard, discomfort, suffering, and pain can be described as an aggregation of low efficacy indicated by long latencies to reach unconsciousness, aversion towards the inhalant gas, induction of fear and anxiety as well as occurrence of distress and pain caused by irritations of the oral and nasal cavity. All these factors can dynamically contribute to the impairment of animal welfare and might act interdependently. Referring to Bali and Jaggi [60], we combined a broad spectrum of methods to assess acute stress, suffering, pain, and discomfort. The methods included behavioral changes, gross inspection and histopathological analyses of the respiratory organs as well as measurement of biochemical markers, such as the stress hormones adrenaline and noradrenaline as well as blood glucose using mice of two strains (NMRI and C57Bl/6J). We focused on the timespan from the first gas exposure to LORR, as during this period animals are thought to be still conscious and, therefore, are able to perceive pain, distress, and anxiety. Attention was paid to practicability and reliability of the three inhalant gases to induce surgical tolerance indicated by the LOPR since this aspect is specifically important for a daily routine.

Overall, the main findings of our study are that high concentrations of CO_2_ (60%, 100% CV/min) and isoflurane (5% with a constant filling rate of 71% CV/min) reliably induced surgical tolerance in both mouse strains within the given timeframe of 300 s. C57Bl/6J mice were more susceptible to isoflurane and sevoflurane than NMRI mice suggesting strain differences in the potency of halogenated ethers. Independent from the strain, behavioral analysis did not reveal signs of distress measured by stress-induced grooming or audible vocalization calls for all inhalant gases during the induction of narcosis. For animals exposed to CO_2,_ we recorded an increase in adrenaline and noradrenaline plasma concentrations compared to Air control as well as compared to isoflurane and sevoflurane, although the effect was strain dependent. In the approach-avoidance test using C57Bl/6J mice, all three inhalant gases were perceived aversive independent from the concentration, and there were no significant differences between the gases. However, in the two groups exposed to isoflurane and sevoflurane some animals became recumbent, whereas recumbency did not occur in animals exposed to CO_2_ in the approach-avoidance tests.

In more detail, CO_2_ 60, CO_2_ 100, and Iso 5% met our requirement of a practicable and reliable anesthetic gas for both strains taking the AD_95_ in pharmacological studies into account [57]. For C57Bl/6J mice, but not for NMRI mice, Sevo 8% can also be rated as an inhalant gas that sufficiently induces narcosis. A primary criterion for euthanasia in terms of animal welfare is that the method achieves rapid unconsciousness [61]. In our study, LORR and LOPR were fastest induced by CO_2_ 100 reflecting a higher potency than isoflurane and sevoflurane administered in high concentrations. In a similar study by Moody et al., shorter timespans for LORR and LOPR were observed in C57Bl/6J mice exposed to CO_2_ and isoflurane [62]. One reason could be the turbulence device we used, which might have caused that the gases did not accumulate as fast at the bottom of the narcotic chamber. In addition, we had to open the cage lid in order to test the LOPR with the forceps. This might have diluted the anesthetic gas concentration in the testing cage with ambient air, and might have resulted in a delayed onset of this reflex. Despite that, the differences in the latencies for LORR and LOPR after CO_2_ and isoflurane exposure of Moody et al. were similar to our observation [62]. Thus, using our experimental setup, the lowest concentrations of CO_2_ (20% CV/min), isoflurane (2%), and sevoflurane (4.8%) cannot be recommended for euthanasia as they did not reliably induce LOPR in both strains. In addition, for NMRI mice 8% sevoflurane occurs also unreliable as less than 90% of the animals per group reached LOPR within 300 s. One could argue that more animals would have reached the LOPR if the experimental time was not limited to 300 s, but we believe that this is the maximum timespan during which animals should reach unconsciousness in a day-to-day routine of euthanasia. From a practical point of view, we cannot recommend the induction of narcosis with CO_2_ at the low filling rate of 20% of chamber volume per min as well as with lower concentrations than 5% for isoflurane and 8% for sevoflurane using our lid construction with turbulence device. This contradicts current euthanasia recommendations implying a filling rate of 20% CO_2_ or gradual filling sufficient to induce LORR and LOPR and, hence, as appropriate methods for euthanizing adult mice [61] and rats [63]. Our recommendation is also in contrast to a recent study by Biovin et al. [64]. They have shown that low CO_2_ chamber replacement rates induce death in C57Bl/6N mice in a sufficient time. As stated above, differences in the experimental setup most likely contributed to the diverging results, i. e. prolonged timespan to reach LOPR. Thus, it is up to a further investigation if CO_2_, isoflurane, and sevoflurane in low concentrations can reliably induce unconsciousness using our experimental setup and longer observation periods.

With regard to animal welfare, it has to be taken into account that a high flow rate of CO_2_ is accompanied by a high concentration in the narcotic chamber at the time of unconsciousness, in our case around 60% for CO_2_ 100. There is great evidence that exposure to already low CO_2_ concentrations is accompanied by fear and anxiety. In humans, exposure to low CO_2_ concentrations of 5% - 7.5% is used as a “CO_2_ challenge test” for anxiety, since a heightened sensitivity to CO_2_ is observed in individuals with a diagnosis of panic disorder [65]. Mice exposed to low CO_2_ concentration (10%) show fear-related freezing behavior and increased anxiety-related behavior in the open field test [66]. In rats, short exposure to rising CO_2_ concentrations (from atmospheric concentrations up to 20% CO_2_) increases anxiety-related behavior and activates brain areas involved in fear and anxiety, mobilizes the HPA stress-axis and initiates stress-related sympathetic responses [67]. The mechanism behind is thought to be the CO_2_-induced pH reduction: by lowering brain pH, pH-sensitive receptors are activated in the fear circuit [68], particularly in the amygdala and locus coeruleus.

Despite these facts, in our study we did not observe corresponding fear- and anxiety-related behavior, such as freezing, increased locomotion, or stress-induced grooming during the induction of narcosis with CO_2_. Mice exposed to CO_2_ occurred rather quiet and appeared sedated and showed a reduced number of rearings. Decreased rearing can be seen as a sign of reduced exploratory and anxiety-like behavior [69, 70], but in our case it is more likely that the reduced vertical activity is related to sedative CO_2_ effects and a sign for beginning muscle weakness at an early stage of narcosis. This assumption is supported by the fact that in both mouse strains CO_2_-induced ataxia and hypotonic gait which are associated with muscle weakness [44]. At first sight, our behavioral observation contradicts the general belief that CO_2_ induces fear and anxiety in mice. However, supporting our results are observations of incidents with CO_2_ in humans. Survivors of CO_2_ poisoning reported, besides a pungent smell and difficulty breathing, no fear, pain, or other warning signs, and unconsciousness was reached within a few seconds [71, 72]. Also, a recent study comparing CO_2_, isoflurane, and pentobarbital-phenytoin euthanasia in mice observed no specific behavioral signs for distress [7].

It can be assumed that mice experience pain analogous to humans when exposed to higher CO_2_ concentrations [65]. CO_2_ has been used as a pain stimulus in animals with similar concentrations that are used for CO_2_ euthanasia [16, 29, 73, 74]. Thus, pain induced by CO_2_ at the mucosa of the nasal and mouth cavity can be assumed. However, also inhalation of isoflurane has been associated with unpleasant sensations. Isoflurane has a pungent smell [75] and it was described that airway irritation and neurogenic constriction are evoked by the activation of TRPA1 channels, which in turn affects the anesthetic induction latency negatively [76]. In humans, 2.3% isoflurane inhalation for one minute provokes coughing, burning, and other unpleasant sensations in the airways in about 40% of subjects, whereas 4% sevoflurane provokes coughing in only 3.7% of the volunteers [33]. Concomitantly, 5% isoflurane induces adverse airway events, as indicated by reflex responses [77]. Analogous to humans, mice can be expected to experience similar sensations at the mucous membranes, such as burning and irritation under isoflurane anesthetic exposure. This has been supposed for rabbits which reluctantly inhale isoflurane and sevoflurane and hold their breath between 30 and 180 s [78]. When analyzing the videotapes, we did not observe any signs for reluctant inhalation or other unpleasant sensations. Unfortunately, we were unable to measure the respiratory frequency, but we noticed that mice exposed to CO_2_ were breathing more deeply following the LORR. Deep breathing could be a sign that those animals moved fast from stage 2 to stage 3, i. e. they reached the stage of surgical tolerance faster than animals exposed to isoflurane or sevoflurane. This assumption is supported by the fact that we observed relatively shorter timespans between LORR and LOPR for CO_2_ than for the two halogenated ethers. We also observed deepening in abdominal breathing in animals exposed to isoflurane and high concentrations of sevoflurane. Deepening of breathing occurred when the animals were already immobile and just before the LORR was measured. Thus, it is most likely that deep breathing in the isoflurane and sevoflurane treatment groups was already a sign for stage 3 of anesthesia. Gross and histologic examination of the respiratory organs revealed no distinct alterations in the three treatment groups that differed from the control group. However, we only detected microscopic atelectasis in mice of both strains when exposed to CO_2_ 60 and 100, but not in mice exposed to halogenated ethers. CO_2_ easily dissolves in blood and has a high diffusion coefficient. Therefore, it is possible that the observed atelectasis is due to reabsorption of CO_2_. Foamy efflux was observed in all animals exposed to isoflurane, sevoflurane, or CO_2_, but only in one control C57Bl/6J mouse. Hence, this seems to be more a specific sign for the inhalant gases. Overall, the results of the gross and histologic examination were incongruent and did not provide a specific sign for pain. This is contradicting a recent study by Biovin et al. that observed more severe pathological alterations of the lungs after CO_2_ exposure compared to isoflurane [7]. But likewise to their conclusion, we cannot determine whether the histological alterations like atelectasis or foamy efflux occurred before the LORR and, hence, were perceived as painful. The recordings of vocalizations provided no further information as we did not detect any in the audible and ultrasound range. In general, mice produce ultrasonic vocalizations during nonaggressive social interactions (for review [47]) and audible vocalizations are expressed when experiencing pain [48]. Valentine et al. observed increased ultrasound vocalization in mice exposed to CO_2_ and isoflurane [41]. However, using this kind of display format, one cannot clearly discriminate between real mouse vocalizations and artefacts, e. g. produced by gas flow.

Concerning the behavioral parameters, the induction of narcosis with isoflurane and sevoflurane increased locomotor activity and led to running excitement, clonus, and opisthotonus before the onset of LORR and, thus, could be interpreted as a stress response. Anesthetic excitement by isoflurane has already been described. It is explained by opposite effects of isoflurane on network excitability in neocortex and hippocampus, which are both involved in anesthetic-induced motor excitation. While low concentrations of isoflurane reduce the excitability in the neocortex, they simultaneously increase it in the hippocampus [79]. Thus, it is difficult to distinguish whether these behaviors reflected distress and/or arousal. In both humans and rodents, unconsciousness is induced by isoflurane already at doses that do not abolish complex movements, and complex movements can be initiated in the spinal cord with minor involvement of supraspinal structures [80]. Running excitement, clonus, and opisthotonus occurred close to the onset of LORR. As we did not see an increase in noradrenaline and adrenaline after exposure to the halogenated ethers, we assume that the excitations were not perceived as stressful.

A valid method to investigate stressful events is the analysis of fast reacting stress hormones such as adrenaline and noradrenaline plasma concentrations as well as blood glucose concentration [50, 81]. In our study, isoflurane and sevoflurane increased blood glucose concentrations compared to CO_2_ and Air control. However, the increase remained within the physiological range [82, 83], thus, we assume that it does not refer to a stress reaction. An explanation of the relative hyperglycemia induced by isoflurane in C57Bl/6J mice is the impaired release of insulin [84]. It is possible that in our study the exposure to the inhalant gases was too short (below 300 s) to induce a so called stress-induced hyperglycemia, which is usually measured 10–15 min after stimulus application [85–87].

With regard to adrenaline and noradrenaline, we observed a marked increase of both catecholamines in response to CO_2_ exposure compared to Air control, although the magnitude differed between the strains with NMRI mice showing higher concentrations than C57Bl/6J mice. Independent from the strain and gas concentrations, both catecholamine concentrations were significantly higher after CO_2_ exposure in comparison to isoflurane or sevoflurane. This increase can be regarded as a solid stress response [88] and suggests that two pathways are involved: on the one hand activation of sympathetic nerve endings by noradrenaline and on the other hand a centrally evoked response by adrenaline [89, 90]. This is supported by findings in pigs, where CO_2_ inhalation before castration or slaughtering leads to large increases in adrenaline and noradrenaline concentrations [91–93] and in humans, where 35% CO_2_ stimulates noradrenaline release 2 min following inhalation and induces subjective feelings of fear [94, 95]. Also in mice, CO_2_ exposure for 15 s leads to an increase in plasma adrenaline and noradrenaline [90]. However, as in our study plasma concentrations of adrenaline and noradrenaline were determined with the onset of LOPR there is a possibility that mice might have already been unconscious at the time of the hormonal stress reaction and therefore have not experienced distress.

Surprisingly, animals exposed to isoflurane and sevoflurane did not respond with an increase in catecholamine concentrations. This could be interpreted that halogenated ethers are not experienced as stressful as CO_2_ by the animals, although a recent study has shown that both isoflurane and CO_2_ lead to a similar increase in the stress hormone ACTH in comparison to a pentobarbital–phenytoin injection [7]. However, in this study ACTH concentrations were measured after the death of the animal and the study does not include a control group. Hence, it is difficult to define if differences in the ACTH concentrations reflect a stress response. In our study, the catecholamine concentrations for the halogenated ethers were even lower than the one of Air control, especially for sevoflurane. This effect might be confounded by the circumstance that the catecholamine concentrations of Air control animals are not equal to baseline levels as the decapitation itself could have caused stress, although we habituated the animals to the fixation procedure [90, 96]. It could also be argued that the missing increase in adrenaline and noradrenaline concentrations after isoflurane and sevoflurane exposure is due to a suppression of stress-related feelings or autonomic reflexes resulting from different signaling mechanisms of isoflurane versus CO_2_. Patch-clamp recordings in murine amygdala slices, a brain area relevant for anxiety, revealed that synaptic signaling by non-NMDA, NMDA, and GABA_B_ receptors is decreased by isoflurane, whereas GABA_A_ receptor-mediated responses are increased [97]. Furthermore, suppression of autonomic reflexes by isoflurane has been demonstrated and explained at least partly by reduced excitability of the amygdala by this mechanism, consequently leading to reduced sympathetic outflow from hypothalamus [97]. Hence, further experiments focusing on anxiety circuits are needed to state clearly that absent elevated adrenaline and noradrenaline levels are equivalent to a lack of fear or anxiety. In addition, studies assessing plasma catecholamines at an earlier stage of anesthesia (at the LORR) and including other fast acting stress hormones like ACTH could provide further insight.

Based on the differences in the catecholamine concentrations between the three inhalant gases we expected to observe a corresponding difference in the approach-avoidance test, i. e. that CO_2_ is perceived significantly more aversive than the two halogenated ethers. It is known that CO_2_ sensing in mice and rats starts at much lower concentrations than those producing trigeminal sensations in humans [98]. Mice detect CO_2_ at near atmospheric concentrations through an olfactory subsystem leading to an innate avoidance behavior [99]. Given the choice, mice leave a chamber gradually filled with low CO_2_ concentrations as 7–12% (our study) and 13.5–18.2% independently of filling rate [52], indicating aversion of mice to these low CO_2_ concentrations. In our approach-avoidance setup, C57Bl/6J mice showed similar escape latencies for all three inhalant gases independent there from the concentrations. Thus, CO_2_, isoflurane, and sevoflurane seemed to be experienced comparably aversive. The results of the second and third exposure have to be interpreted with caution as it was shown for isoflurane and sevoflurane that re-exposure can lead to increased aversion in rodents [13, 22, 100, 101]. Here, a separate analysis of the initial exposure did not reveal a significant difference in the escape latencies for CO_2_, isoflurane, and sevoflurane contradicting previous findings using similar experimental setups and including a single gas exposure. Leach et al. observed that CO_2_ was the most aversive gas compared to several halogenated ethers in mice, and also Moody and Weary found isoflurane to be more aversive than CO_2_ indicated by longer dwelling times [13, 15]. A more recent study using a new aversion test revealed sevoflurane to be the least aversive inhalant gas compared to isoflurane and CO_2_ [35]. However, we also noted that some animals of the groups exposed to isoflurane or sevoflurane, but not CO_2_, became recumbent arguing for a higher aversiveness of CO_2_. A similar observation was made for mice and rats which became recumbent when exposed to isoflurane or sevoflurane, but not to CO_2_ [13, 52, 101]. However, recumbency mostly occurred in the groups re-exposed to halogenated ethers and only one animal of the isoflurane group became recumbent during the first exposure. Thus, the lacking significant differences in the approach-avoidance test during the first gas exposure seem to correlate with the missing differences in the behavioral parameters of our first experiments.

To sum up, it cannot be decided yet which of the inhalant gases is the best option to euthanize laboratory mice. It needs an ethical discussion whether a longer procedure with less maximum stress counterbalances a shorter procedure with more maximum stress. Despite all the new data of our present study, it is still difficult to weigh up the advantages and disadvantages of CO_2_ against isoflurane and sevoflurane narcosis. All three gases were sensed aversive by the animals. The seemingly rather quiet behavior during CO_2_ exposure as opposed to the more ‘agitated’ behavior during isoflurane exposure may be misleading in the interpretation of stress. We argue that the behaviors evoked by isoflurane are rather due to the typical excitement phase of inhalant anesthetics and are accompanied by the loss of consciousness. The marked increase in adrenaline and noradrenaline evoked by CO_2_ indicates a higher stress response compared to isoflurane and sevoflurane. However, the suppression of autonomic reflexes by the halogenated ethers may lead to a missing catecholamine increase. Measurement of other fast reacting stress hormones like ACTH might be helpful to confirm our findings. Sevoflurane showed a strain-dependent efficacy and might not be sufficient for euthanasia of all mouse strains. It also needs to be clarified, how the different parameters of this study are affected by CO_2,_ isoflurane, or sevoflurane, if several animals are euthanized at the same time in one home cage. Still, our data suggests that rather high concentrations of halogenated ethers shall be applied and low concentrations of CO_2_ should be omitted. Our results do not provide enough evidence that CO_2_ can be fully replaced by isoflurane and sevoflurane to kill laboratory rodents.

## Supporting information

S1 Supporting Information Raw datasets including the latencies to LORR and LOPR, the occurrence of rearings, ataxia, and locomotion, the occurrence of convulsions, stress-induced grooming, running excitement, and opisthotonus, vocalizations, glucose, adrenaline, and noradrenaline concentrations, gross examination and histology of the respiratory tract, and escape latencies from the narcotic chamber of the approach-avoidance test. (XLSX)

## Abbreviations

ACTH: adrenocorticotropic hormone
ASIC: acid sensing ion channel
CO_2_: carbon dioxide
CV: chamber volume
HPA: hypothalamic-pituitary-adrenal
HPLC: high pressure liquid chromatography
Iso: isoflurane
LOPR: loss of the pedal withdrawal reflex
LORR: loss of the righting reflex
NaCl: sodium chloride
Sevo: sevoflurane
